# Advanced Fabrication of Chemically Bonded Graphene/TiO_2_ Continuous Fibers with Enhanced Broadband Photocatalytic Properties and Involved Mechanisms Exploration

**DOI:** 10.1038/srep38066

**Published:** 2016-12-01

**Authors:** Qingzhe Zhang, Nan Bao, Xinqiang Wang, Xinde Hu, Xinhan Miao, Mohamed Chaker, Dongling Ma

**Affiliations:** 1 School of Environmental Science and Engineering, Shandong Key Laboratory of Water Pollution Control and Resource Reuse, Shandong University, Shanda South Road 27, Jinan 250100, P. R. China; 2 Institut National de la Recherche Scientifique (INRS), Centre Énergie Materiaux et Télécommunications, Université du Québec, 1650 Boulevard Lionel-Boulet Varennes, Québec J3X 1S2, Canada; 3 State Key Laboratory of Crystal Materials, Shandong University, Shanda South Road 27, Jinan 250100, P. R. China

## Abstract

In this article, a novel route for the synthesis of graphene/TiO_2_ continuous fibers (GTF) using force-spinning combined with water vapor annealing method is reported for the first time. The morphology, structure and optical properties of the composite were fully characterized. With a single step of heat treatment process using steam at ambient conditions, we were able to initiate a series of chemical reactions, such as reduction of graphene oxide (GO), crystallization of TiO_2_, formation of C-Ti bond, and introduction of oxygen vacancies into TiO_2_. The incorporation of graphene in TiO_2_ fibers facilitated bandgap narrowing and improved photo-induced charge separation in the photocatalyst. As a result of synergistic effects, TiO_2_ fibers-2 wt% graphene (2%GTF) showed the highest photocatalytic activities in the degradation of X-3B under UV irradiation, superior to the benchmark photocatalyst P25. Under visible light irradiation, the same catalyst was about 4 times more efficient compared to pure TiO_2_ fibers (PTF). A detailed study of involved active species (in particular, ·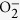, h^+^ and ·OH) unraveled the mechanism regarding photocatalysis.

As a non-polluting, widespread and inexhaustible resource, solar energy has long been considered as one of the most promising renewable energy sources in the world^1^,^2^,^3^. Regarding solar energy utilization, heterogeneous photocatalysis has appeared as an efficient method in the field of environmental remediation^4^. Nonetheless, it remains one of the greatest challenge to find suitable photocatalysts that can harvest the wide spectrum of solar light, from Ultra Violet (UV) to visible, and achieve efficient solar energy conversion^5^,^6^. TiO_2_ has been widely studied in photocatalytic degradation of contaminants through harvesting solar energy, due to its biological and chemical inertness, cost effectiveness, nontoxicity and relatively high activity^1^,^7^,^8^. However, the high recombination possibility of the photo-generated electrons and holes on the surface and in the bulk phase of the TiO_2_ leads to a low quantum yield^8^. Moreover, the photocatalytic activity of TiO_2_ is mainly confined to the UV light (λ < 380 nm), which accounts for only ~4% of the incoming solar energy because of its wide bandgap (3.0–3.2 eV). In addition, the poor adsorbing affinity of TiO_2_ towards target pollutants causes the low photocatalytic efficiency. Up to now, a variety of strategies, including metallic^9^ and nonmetallic doping^10^, noble-metal loading^10^,^11^, as well as metal oxide^12^,^13^ and metal hydroxide loading^14^, have been employed to enhance the photocatalytic performance of TiO_2_. In particular, introducing carbonaceous nanomaterials (such as activated carbon and carbon nanotubes)^15^,^16^,^17^ into TiO_2_ represents one of the most efficient approaches to improve the photocatalytic activity.

Graphene, known as a single-layered sheet of sp^2^-hybridized carbon atoms, has attracted increasing research interest owing to its superior properties in capturing and transporting electrons, large specific surface area, high transparency and strong adsorption capacity^18^,^19^,^20^,^21^. Recently, the incorporation of graphene into TiO_2_ has gained widespread attention, because it could greatly enhance the activities in photodegrading pollutants^22^. However, most of these studies have been mainly conducted on the combination of graphene with nanosized TiO_2_ powders which tend to aggregate in suspension, prohibiting the photoelectron injection from TiO_2_ into graphene, causing a rapid loss in active sites and thereby photocatalytic activity. In addition, the difficulty in post-separation and recycling of photocatalysts in the slurry system poses a key obstacle to their practical application.

Compared with the nanoparticle photocatalysts, one-dimensional fibers possess high surface area per unit volume^23^, excellent mobility of charge carriers^24^, and the ability in harvesting more light at the red part of the solar spectrum^25^. Electrospinning is a common and traditional method in the preparation of nanofiber photocatalysts^25^. In this method, polymer templates are typically used to improve the viscosity of precursor solution to make the fibers form. However, with the use of polymers most of the active sites for photocatalysis are located inside the fibers, instead of on the surface, which considerably restricts the light utilization of photocatalysts. K. Sarkar *et al*.^26^ demonstrated that the fibers fabricated by electrospinning have large standard deviations in diameters and low purity because of the potential residue of polymer beads involved in the processing. Furthermore, the reduction of graphene oxide (GO) mainly relies on chemical method, which requires strong reducing agents, and the reaction conditions are usually vigorous, uncontrollable and not environment friendly.

Herein, a centrifugal force spinning method, without high electric fields and any template or binder polymer, was employed for the first time to synthesize the precursor fibers of GO/TiO_2_. Then these precursor fibers with different GO content were annealed at 500 °C under water vapor to prepare graphene/TiO_2_ continuous fibers (denoted as xGTF, where x is the GO content by mass). Subtly using the water-gas reaction during the calcination to reduce GO, the C-Ti chemically bonded xGTF with self-doping of Ti^3+^ and oxygen vacancies was obtained. The main reactions in the process of calcination were analyzed briefly. The xGTF photocatalysts exhibited exceptional photocatalytic reactivity in degrading reactive brilliant red X-3B, a refractory typical azo dye, in wastewater compared with pure TiO_2_ fibers (PTF) and benchmark photocatalyst P25 under both UV and visible light irradiation. The morphology, structure and optical properties of fibers were thoroughly characterized by various techniques, and the photocatalysis mechanisms were explored by detecting the radicals involved in the reactions.

## Results and Discussion

### Structure and morphology characterizations

#### Morphology and Crystal Structure

**Figure 1:**
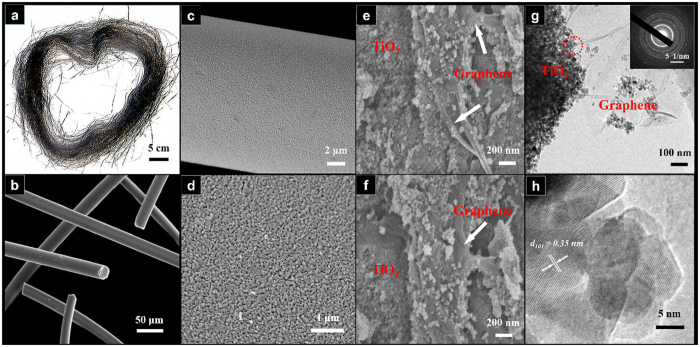
Photo (**a**), SEM (**b–f**), typical TEM (**g**) and HRTEM (**h**) images of 2%GTF. The inset in (**g**) is SAED pattern and (**h**) is corresponding to the area encircled in (**g**).

The photo of 2%GTF is shown in Fig. 1a, which demonstrates that the prepared fiber photocatalysts are continuous and long, almost reaching several meters in length that make them easy to be separated and recycled from the post slurry during wastewater treatment. Centrifugal force spinning overcame many problems frequently encountered during electrospinning, such as the tendency of fibers to cling together, uneven diameters and low purity of fibers^26^, *etc*. The field-emission scanning electron microscopy (FESEM) image (Fig. 1b) reveals that the diameters of the fibers were quite uniform and about 20 μm. All the fibers were separated with each other which facilitated the post processing and applications. The fiber photocatalysts were composed of dense TiO_2_ nanocrystals (Fig. 1c and d) with graphene embedding into them (Fig. 1e and f). The rough surface structure was favorable for adsorption and subsequent photodegradation of organics in wastewater. The transmission electron microscopy (TEM) image of 2%GTF confirmed the coexistence of TiO_2_ and graphene which was demonstrated to be a transparent, smooth and layered material (Fig. 1g). The high resolution TEM (HRTEM) image shown in Fig. 1h exhibits a lattice fringe of 0.35 nm, corresponding to the (101) crystallographic plane of anatase TiO_2_. A selected-area electron diffraction (SAED) pattern (inset of Fig. 1g) showed the multi-crystalline feature of TiO_2_ crystals.

**Figure 2:**
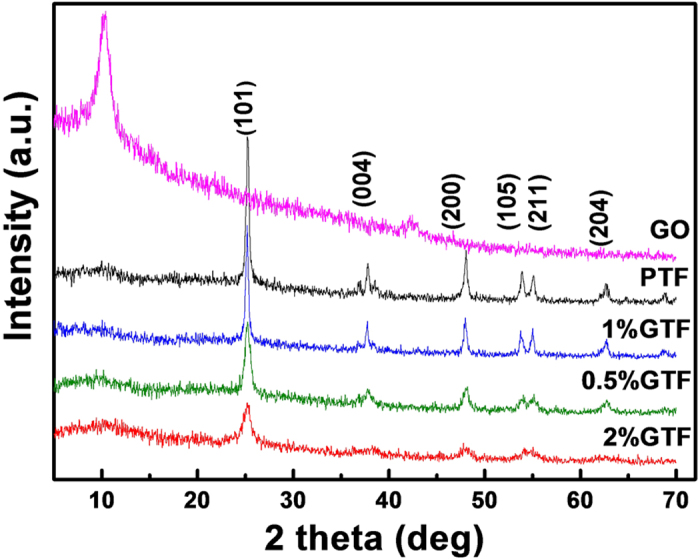
XRD patterns of GO, PTF and xGTF (x = 0.5, 1, 2%) composites.

The X-ray diffraction (XRD) patterns provide information on the crystalline nature of GO, PTF, and xGTF hybrid materials and are presented in Fig. 2. In the case of GO, the peak at 2 theta of 10.4° indicates that graphite has been considerably oxidized into GO with significantly expanded interlayer distance from 0.335 nm to 0.848 nm. The large d-spacing of GO was related to the oxygenic functional groups on carbon backbones, such as hydroxyl, carboxyl and epoxide groups, *etc*.^27^. The XRD patterns of xGTF were similar to that of PTF, which corresponded to the pure anatase phase of TiO_2_ (JCPDS No. 21–1272), in good agreement with the analysis of HRTEM image. Notably, no characteristic diffraction peaks for GO appeared in the composite, which may be due to the low amount and relatively low diffraction intensity of graphene, or GO was probably reduced to graphene by the annealing under water vapor.

The crystallite diameters estimated by using the Scherrer equation are listed in Table 1. According to the results, the grain size of TiO_2_ decreased with the increase of graphene content. It shows that the TiO_2_ grain growth was confined with the introduction of graphene, similar to that shown in the report of Gao^28^, which could be attributed to the inhibiting effect of graphene sheets.

#### FT-IR Analysis

**Figure 3:**
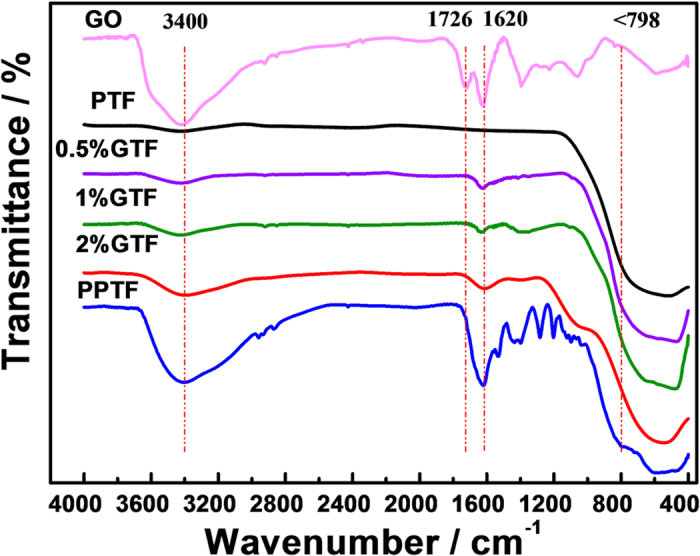
FT-IR spectra of GO, PPTF, PTF and xGTF (x = 0.5, 1, 2%).

The Fourier transform infrared (FT-IR) spectra of GO, precursor of PTF before annealing (PPTF), PTF and xGTF are displayed in Fig. 3. A broad and strong peak at ~3400 cm^−1^, assigned to the stretching vibration of hydroxyl groups, was observed for all the samples, indicating the presence of undissociated H_2_O molecules (H-bond *ν*_OH_) adsorbed on the surface samples^29^ or surface hydroxyl (−OH) groups^23^. It was reported by Linsebigler *et al*.^30^ that surface −OH groups could act as electron donors of generated H^+^, accept photo-induced holes and produce ·OH radicals which are very helpful in improving the photocatalytic activity. The broad absorption at low frequency (below 798 cm^−1^) was attributed to the vibration of Ti-O-Ti and C-O-Ti^23^, which confirmed the formation of TiO_2_ and C-O-Ti bond in xGTF. The spectrum of PPTF clearly shows many absorption bands, including the C-OH stretching at 1368 cm^−1^, C-H bending vibration at 1456, 1154, 1089 and 1030 cm^−1^, and C-CH_3_ stretching at 1290 cm^−1^. These absorption bands were not pronounced in PTF, indicative of an apparent removal of organics in PPTF after the calcination under steam.

The absorption band at 1620 cm^−1^ was attributed to skeletal C=C vibration in graphene^31^ which corresponded to the sp^2^ character and the C-O stretching^32^. In the spectrum of GO, the characteristic peaks of GO at 1726 cm^−1^, corresponding to the C=O carbonyl stretching, and at 1050 cm^−1^ due to the C-O stretching were observed^33^. Both bands were not found in other samples, including the xGTF, suggesting the removal of oxygen-containing groups and the reduction of GO during the water vapor annealing. The reduction of GO could enhance the electrical conductivity of graphene, and improve the transfer efficiency of photo-induced charges.

#### XPS Measurement

**Figure 4:**
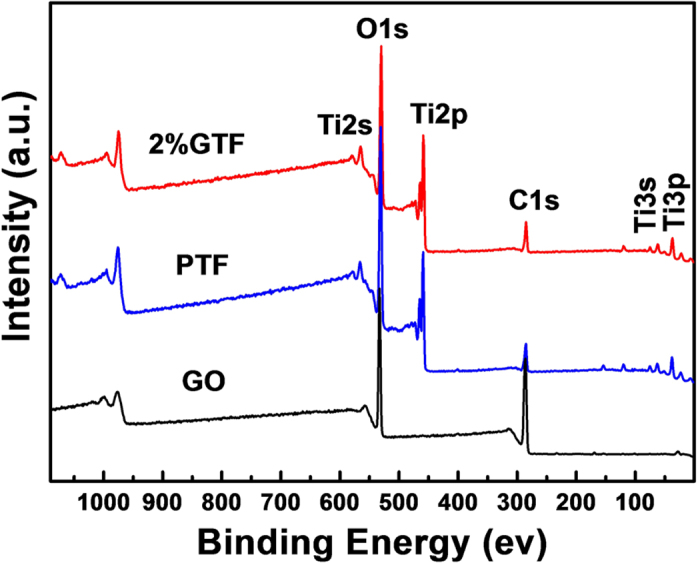
XPS survey spectra of 2%GTF, PTF and GO.

The interactions between graphene, TiO_2_ and functional groups as well as the chemical state of all the elements were analyzed by X-ray photoelectron spectroscopy (XPS). All the obtained data were calibrated by using contaminant carbon at binding energy of 284.7 eV. The XPS survey spectra of 2%GTF, PTF and GO are shown in Fig. 4. It was noticed that 2%GTF only contains Ti2p, O1s and C1s peaks, indicating it consisted of C, Ti and O elements without any other detectable impurities.

**Figure 5:**
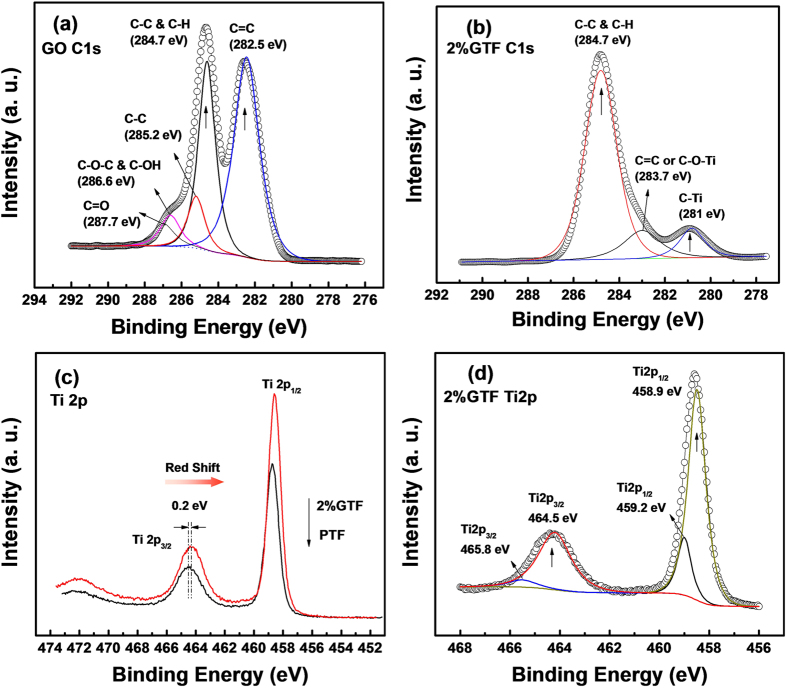
Peak deconvolution of the C(1s) XPS core level of GO (**a**) and 2%GTF (**b**), XPS spectra of Ti2p for PTF and 2%GTF (**c**) and peak deconvolution of Ti2p for 2%GTF (**d**).

As presented in Fig. 5a, deconvolution peaks of the C1s spectrum suggested the abundance of oxygen-containing groups on the surface of GO. The peak at 284.7 eV (C-C bond) and 282.5 eV (C=C) bond were mainly assigned to the sp^2^ hybridized graphitic carbon atoms^34^,^35^. The observed peak at 285.2 eV was attributed to sp^3^ hybridized carbon^36^. While two peaks located at the binding energies of 286.6 and 287.7 eV were assigned to oxygen bound species of C-O-C of epoxy group^36^,^37^,^38^, and C=O of carbonyl or carboxyl^36^,^39^ on the surface of GO, respectively.

Figure 5b shows the C1s XPS spectrum of 2%GTF. The disappearance of peaks corresponding to C=O and C-O-C groups indicated that GO in the composite was reduced after the heat treatment with water vapor activation, which coincided with the FT-IR results. The two peaks centered at 284.7 and 283.7 eV corresponded to the C-C and C=C bonds^34^, respectively. The peak around 283.7 eV could also be contributed by C-O-Ti bond^40^,^41^. Notably, the additional peak at the binding energy of 281 eV appeared and was ascribed to C-Ti carbide bond^34^. Along with the C-O-Ti bonds, they strong suggest the existence of chemical binding between TiO_2_ and graphene^34^. The formation of such binding between TiO_2_ and graphene is expected to be quite advantageous for the desired, efficient transport of charge carriers through the composite upon light excitation to greatly enhance its photocatalytic activity^36^,^39^. The formation of C-O-Ti and C-Ti bonds could also broaden the light absorption to the visible light region^36^.

The XPS Ti2p core level spectra of PTF and 2%GTF are displayed in Fig. 5c. As for the PTF, two characteristic peaks centered at 464.5 and 458.9 eV were attributed to Ti2p_1/2_ and Ti2p_3/2_ spin-orbital splitting photoelectrons, respectively^42^,^43^. The chemical shift of Ti2p_1/2_ and Ti2p_3/2_ was typically 5.6 eV, which referred to the presence of the normal state of Ti^4+^ in the as-prepared photocatalysts^43^. With respect to Ti2p XPS spectrum of 2%GTF, a red shift of 0.2 eV to the lower binding energy was observed, indicating the appearance of Ti^3+^ in 2%GTF^42^. The cause of the Ti^3+^ formation is as follows. On the surface of TiO_2_, free carbon tends to react with oxygen in the air, while in an anoxic water vapor atmosphere, it favors oxygen in the lattice of TiO_2_, leading to the formation of oxygen vacancies (O_v_) and the low valence state of Ti^3+ ^42,44. The O_v_ and Ti^3+^ could act as electron traps and inhibit the recombination of photo-induced e^−^ and h^+^ to improve the photocatalytic activity. The peak deconvolution of the Ti2p XPS spectrum of 2%GTF revealed two additional weak peaks located at 465.8 and 459.2 eV. They are related to the Ti2p_1/2_ and Ti2p_3/2_ spin-orbital splitting photoelectrons from C-Ti bonds (Fig. 5d), which confirmed the formation of chemical bond^34^,^43^ between TiO_2_ and graphene in 2%GTF.

#### Raman Analysis

**Figure 6:**
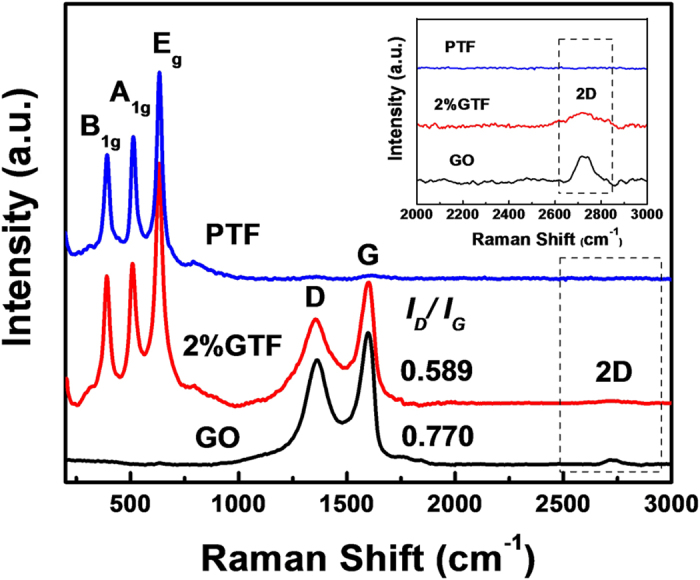
Raman spectra of PTF, 2%GTF and GO, with the 2D band region enlarged in inset.

Raman and FT-IR spectrum analysis complement each other and were used to analysis the structure and composition of materials by detecting molecular vibration. The Raman spectra of PTF, 2%GTF and GO are shown in Fig. 6.

In the Raman spectra of PTF and 2%GTF, sharp peaks around 399 cm^−1^ (B_1g_), 513 cm^−1^ (A_1g_) and 639 cm^−1^ (E_g_) were all ascribed to anatase TiO_2_, which was in agreement with the XRD and HRTEM analysis results. The characteristic D band (1350 cm^−1^) and G band (1595 cm^−1^) were observed in the Raman spectra of both 2%GTF and GO. The intensity ratio of the D and G bands, documented as I_D_/I_G_, was used to characterize the relative concentration of local defects, particularly the sp^3^-hybridized defects, or disorders with respect to all sp^2^ carbon atoms including the sp^2^-hybridized graphene domains^35^,^45^. From the Raman spectrum analysis, the I_D_/I_G_ ratio of GO was found to be 0.770. Importantly, this ratio was decreased to 0.589 in 2%GTF, indicating the reduction of GO^34^,^35^,^45^,^46^ during the process of heat treatment under water vapor annealing. The decrease of I_D_/I_G_ ratio indicated that the density of defects and disorders was reduced, more carbon atoms were graphitized and the π-π conjugation was greatly reconstructed in 2%GTF. With no doubt, such reconstruction will facilitate the transfer of e^−^ from TiO_2_ to graphene and suppress the recombination of e^−^/h^+^, highly beneficial to photocatalysis. In addition, the position of D and G bands red shifted, confirming the reduction of GO once again^35^,^47^. In addition, the 2D band at around 2780 cm^−1^ originated from two phonon double resonance^23^, appeared in the Raman spectra of 2%GTF and GO (Fig. 6, inset). Being different from graphite, the relatively symmetric 2D band demonstrated that the graphite has been successfully exfoliated to layered graphene and introduced into 2%GTF composite^48^. Moreover, the line shape of the 2D band can be used to determine the number of graphene layers. To do that, the 2D peaks of 2%GTF and GO measured under 514 nm laser excitation were compared with the results reported by Graf *et al*.^49^ and Ferrari *et al*.^50^, and it was identified that the as-prepared graphene possesses 2–3 layers.

#### Textural Properties Test

Photocatalysis is essentially a surface reaction, mainly taking place on the surface of catalysts rather than in the bulk. Therefore, the adsorption and pre-enrichment of pollutants onto the photocatalysts surface is the prerequisite step. The texture of materials play a very important role in their adsorption capability^29^,^51^. Table 2 shows the main textural parameters of xGTF and PTF, including the BrunauerEmmet-Teller (BET) surface area, pore volume and pore diameter, *etc*.

**Figure 7:**
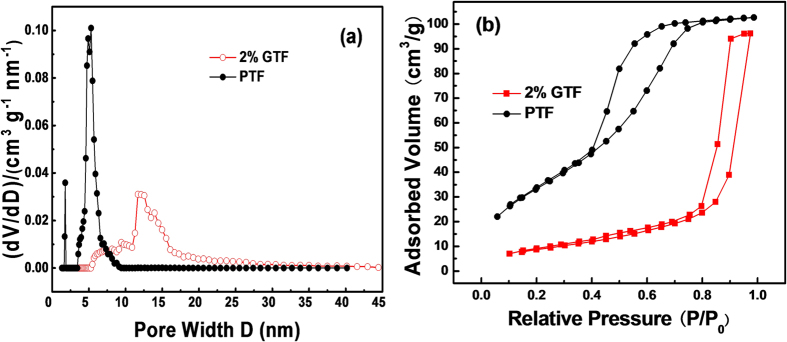
Distribution of pore size (**a**) and adsorption isotherms (**b**) of PTF and 2%GTF.

From Table 2, it can be seen that with the increase of GO content, the BET surface area, pore volume and pore diameter of composites were improved greatly. The introduction of graphene enhanced the texture properties of photocatalysts, and 2%GTF possessed the biggest BET surface area of 86.772 m^2^/g, almost twice that (44.834 m^2^/g) of PTF. Mean pore diameters of all the samples were about 3–12 nm in the mesoporous range of 2–50 nm. Pore structures of 2%GTF and PTF were investigated by analyzing the pore size distribution curves (Fig. 7a) and adsorption isotherms (Fig. 7b).

As shown in Fig. 7a, the most probable pore sizes of 2%GTF and PTF were around 11 and 3.6 nm, respectively, indicating that the samples were mesoporous structures. The peak of PTF curve was much sharper than that of 2%GTF, demonstrating that the introduction of graphene gave rise to the irregular pore size distribution.

The types of N_2_ adsorption-desorption isotherms and hysteresis loops were classified by the nomenclature of Brunauer-Deming-Deming-Teller^52^ and International Union of Pure and Applied Chemistry. The adsorption isotherms of 2%GTF and PTF were both approximately categorized as a type IV isotherm with a type H2 hysteresis loop (Fig. 7), which were usually attributed to capillary condensation that occurs in the mesopores, indicating the presence of mesoporous structures with narrow pore distribution. Type H2 hysteresis loop meant that the as-prepared materials possessed complex pore structures, including the ink-bottle-shaped pores with narrow and wide sections, and possible interconnecting channels. In addition, the areas of hysteresis loops were relatively large, reflecting the presence of regular pore structures^53^.

### Heat Treatment Process under Steam Ambient

**Figure 8:**
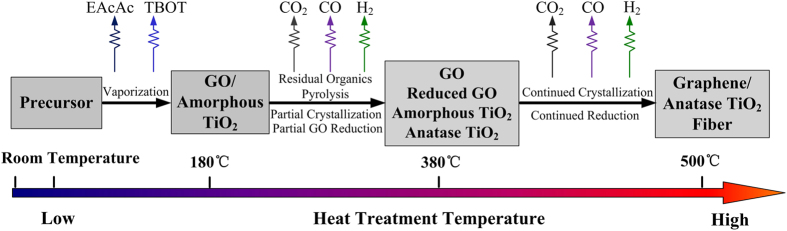
The heat treatment process of fibers in the tube furnace under steam ambient.

The heat treatment and activation process of precursor fibers were illustrated in Fig. 8, containing multiple heterogeneous reactions. The whole process consisted of three stages.

In the first stage, most of the organics in the precursor, including EAcAc and TBOT, evaporated in forms of molecules in the temperature range of 20–180 °C. GO and amorphous TiO_2_ were main components of the precursor fibers. Under the ambient of water vapor, further hydrolytic polycondensation of polymers in the precursor took place to form Ti-O-Ti and some big pores formed on the surface. With the further increase of temperature from 180 °C to 380 °C, some leftover organics would be thermally decomposed in the second stage. The residual carbon reacted with water vapor, producing CO and H_2_, and then a fraction of TiO_2_ would react with the produced CO to generate CO_2_ and Ti_2_O_3_ with the colors of blue or purple. These reactions can be described by the following Equations (1)–(2).


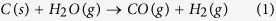



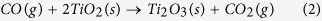


In this second stage, TiO_2_ was partially crystallized into the anatase phase and GO was partially reduced by reacting with the produced H_2_ as well. In the last stage, higher temperature facilitated the further crystallization of TiO_2_ and the production of more H_2_, which could efficiently reduce the remaining oxygen-containing functional groups on GO. In the process of reduction of GO, C-Ti bonds formed between TiO_2_ and graphene, and moderate Ti^3+^ and oxygen vacancies emerged on the surface of the composite photocatalysts. The mechanism of the formation of C-Ti bonds was proposed as follows. Before the production of H_2_, the -COOH groups on the GO surface possibly react with the −OH groups on the nanocrystalline TiO_2_ through esterification to produce O=C-O-Ti bonds^41^ (Equation (3)). Hence, the oxygen-containing functional groups on the GO played a great role in this thermal treatment process. After producing CO and H_2_, the C-O-Ti bonds may be transformed into C-Ti bonds under the reductive atmosphere at a reasonable annealing temperature (500 °C, Equation (4)).


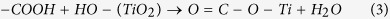



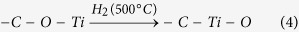


Interestingly, in the single process, the removal of organics, the crystallization of TiO_2_, the reduction of GO, the formation of C-Ti bonds, and the self-doping of Ti^3+^ and the creation of oxygen vacancies were achieved at the same time.

### Optical Characterizations

#### UV-vis DRS Measurement

**Figure 9:**
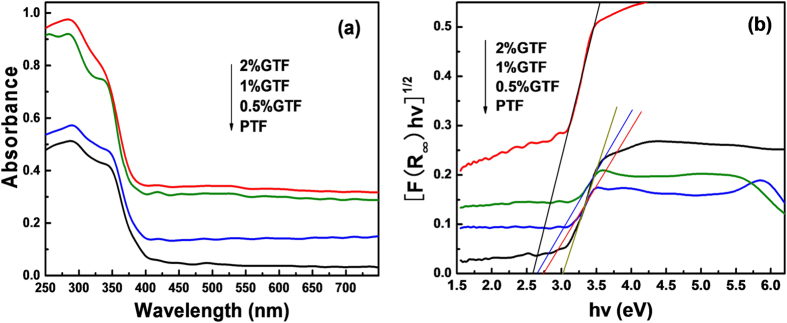
UV-visible diffuse reflectance spectra (DRS) of PTF and XGTF (x = 0.5, 1, 2%) (**a**), and the plot of transformed Kubelka-Munk function versus the energy of light (**b**).

As stated above, the light absorption range of photocatalysts was of great importance in photocatalysis, especially for the visible light photodegradation of organic pollutants in wastewater. The UV-vis diffuse reflection spectra (DRS) of as-prepared PTF and xGTF composites are presented in Fig. 9a.

Based on the analysis of DRS, stronger absorption can be achieved in both UV and visible regions for xGTF, compared to PTF photocatalysts. 2%GTF exhibited the highest absorption intensity in these regions. Meanwhile, red shifts of the absorption edge were observed. Basically, with the increase of graphene content, the extent of red shifts increased. It suggests that the introduction of graphene could narrow the bandgap of TiO_2_ photocatalysts, which was attributed to the formation of C-Ti bond^54^,^55^. From the plot of transformed Kubelka-Munk function (Fig. 9b), bandgaps were narrowed from 3.02 eV of PTF, to 2.74 eV of 0.5%GTF, 2.65 eV of 1%GTF, and 2.59 eV of 2%GTF. Bandgap narrowing of as-prepared photocatalysts made them possess a broader spectral response in the visible light region, highly relevant to visible photoactivity.

#### PL Spectra Analysis

**Figure 10:**
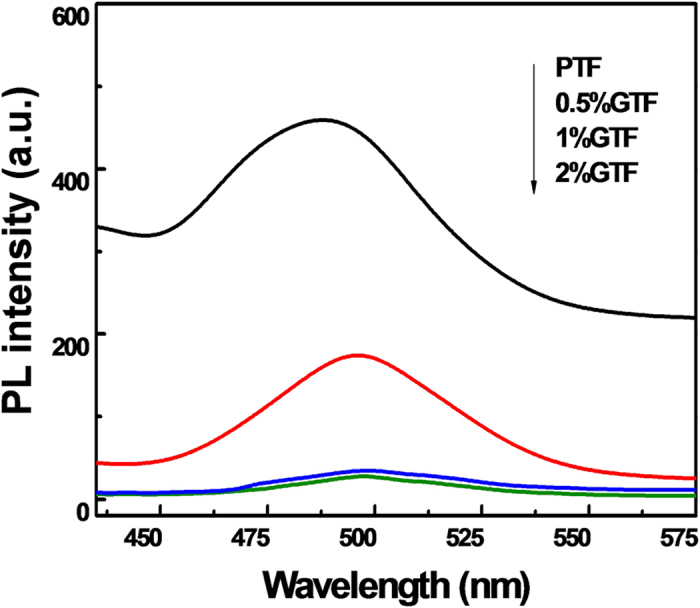
Photoluminescence spectra of PTF and xGTF (x = 0.5, 1, 2%).

The PL emission spectra have been widely employed to study the excited state of semiconductors and to understand the fate of photo-induced e^−^/h^+^ pairs, including the trapping, immigration and transfer of charge carriers^56^. When charge carrier recombination took place in photocatalysts under irradiation, the extra energy could be released by emitting photons, resulting in PL. The PL spectra of xGTF and PTF excited at 375 nm are presented in Fig. 10.

As shown in Fig. 10, PTF exhibited a broad emission band with the peak position at ~500 nm, corresponding to the emission in the anatase TiO_2_^57^, which was in accordance with the results of XRD and Raman analysis. With the increase of graphene content, the intensity of the emission band weakened. The dramatic emission quenching was observed in xGTF (x = 1 and 2%), indicating that electrons were largely trapped by defect sites, such as Ti^3+^ and oxygen vacancies, or efficiently transferred from the conduction band of TiO_2_ to graphene prior to recombination^25^. The transfer of electrons may be explained by the following reasons. With respect to xGTF composites, a heterojunction, space-charge separation region, formed at the interface of TiO_2_ and graphene. Electrons tended to flow from the higher (TiO_2_) to lower (graphene) Fermi level^56^,^58^. So the introduction of graphene could accept photo-induced electrons from TiO_2_, thus suppressing the recombination of charge carriers. Furthermore, according to the result of XPS, the C-Ti covalent bond formed between TiO_2_ and graphene could facilitate the charge transfer, resulting in a lower recombination rate of carriers and enhanced photoactivity.

### Enhanced Photocatalytic Activity with Broad Spectral Response

**Figure 11:**
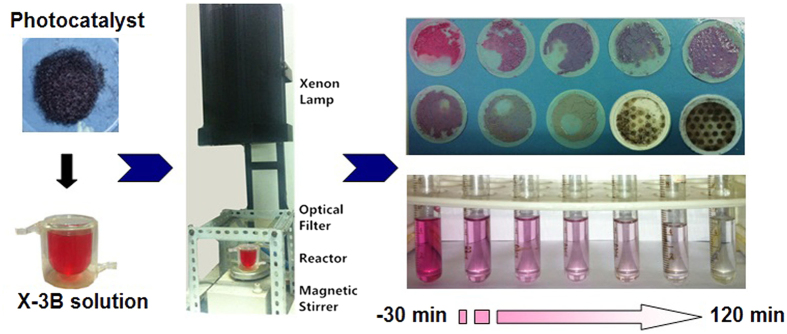
The schematic illustration of photodegradation of X-3B by the as-prepared photocatalysts under visible-light.

The photocatalytic performance of the as-prepared PTF and GTF photocatalysts were evaluated by degrading X-3B in solution as a model reaction under the separate irradiation of UV and visible lights (λ > 420 nm). Figure 11 shows the schematic illustration of the photocatalytic reaction under visible light.

**Figure 12:**
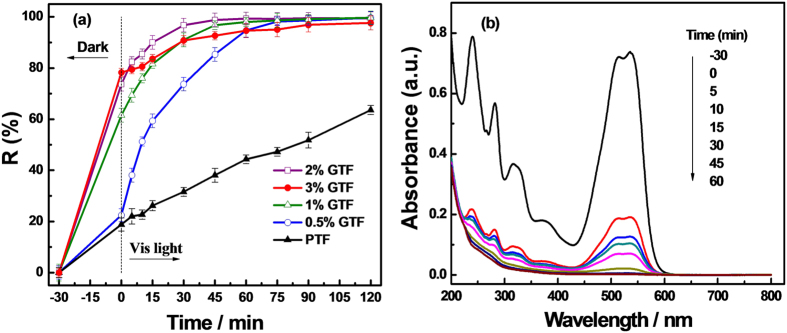
Photodegradation of X-3B by PTF and xGTF (x = 0.5, 1, 2%) under visible light (**a**) and UV-Vis absorption spectra of X-3B degraded by 2%GTF (**b**).

The photodegradation of X-3B by PTF and xGTF and UV-Vis absorption spectra of X-3B photodegraded by 2%GTF are displayed in Fig. 12. As shown in Fig. 12a, after adsorption equilibrium in the dark for 30 min, the adsorptivity towards X-3B enhanced greatly with the increase of graphene content in as-prepared photocatalysts. A little more X-3B was adsorbed onto 3%GTF than 2%GTF. The adsorption rate of 3%GTF towards X-3B almost reached 78%, while PTF only reached 20%. The enhanced adsorptivity was attributed to the highly developed porous structure and large surface areas of the materials caused by the introduction of graphene, which was supported by the textural property analysis. In addition, X-3B molecules could be adsorbed onto the surface of xGTF, with offset face-to-face orientation via π-π conjugation between the aromatic regions of the graphene and dyes^36^,^37^,^54^, and therefore, the adsorptivity was enhanced compared to bare PTF.

As a prerequisite for photocatalytic reaction, the enhanced adsorptivity of photocatalysts would facilitate the photodegradation of X-3B. As shown in Fig. 12, after 30 min visible-light irradiation, it was clearly observed that 2%GTF exhibited the best photocatalytic activity towards X-3B among all the prepared samples, with the photodegradation efficiency of 98%, being ~4 times that of PTF. According to the above characterization and analysis, except for the strong adsorptivity, the enhanced photocatalytic activities could be explained by the following reasons. The reduction of GO achieved the maximum restoration of π-π conjugation in the graphene plane to promote the electrical conductivity of graphene. The restoration of π-π conjugation, the formation of C-Ti bond, the presence of Ti^3+^ and oxygen vacancies would facilitate the separation and suppress the recombination of photo-induced e^−^/h^+^ pairs, as supported by the analysis result of PL spectra. Furthermore, the bandgap of photocatalyst was narrowed by forming the C-Ti bond, making it possible to respond to the visible light and show improved visible photocatalytic activity. The photoactivity of 3%GTF was even lower than 1%GTF. The reason may be that excessive addition of graphene would lower the light intensity through the depth of reaction solution^45^, or excessive graphene in photocatalysts increased the opportunity for the collision of e^−^ and h^+^ and promoted the recombination of the photo-induced carries^42^. The UV-vis absorption spectra are illustrated in Fig. 12b. The characteristic absorption band of X-3B around 540 nm decreased drastically during adsorption in the dark for 30 min. Subsequently after 45 min of the visible-light irradiation, the characteristic peak almost completely disappeared, indicating the X-3B in solution was nearly fully photodegraded.

**Figure 13:**
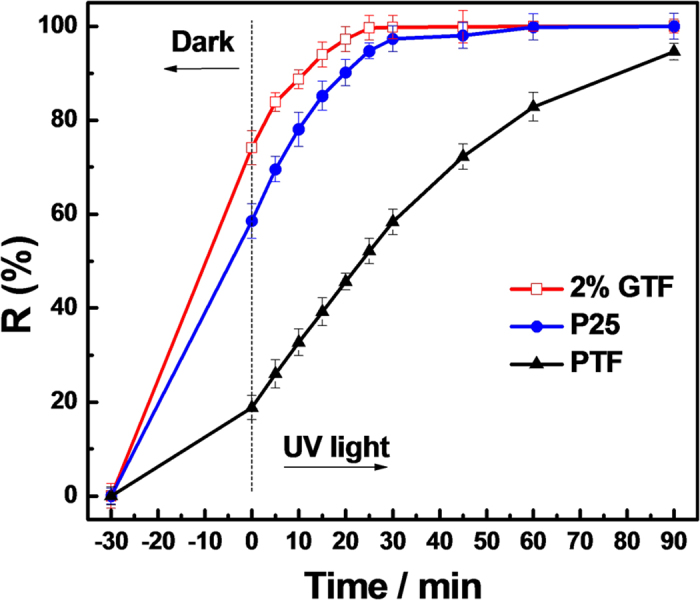
Photodegradetion of X-3B by 2%GTF, P25 and PTF under UV light irradiation.

The photodegradation of X-3B by 2%GTF, P25 and PTF under UV light is displayed in Fig. 13. 2%GTF exhibited enhanced photocatalytic properties compared to commercial P25 under UV light, and the photocatalytic activity decreased in the order of 2%GTF > P25 > PTF. So 2%GTF still maintained the superiority in activity under UV-light irradiation. Thus, the as-prepared composite photocatalysts possessed enhanced broadband photocatalytic activities.

### Detection of active species and proposed photocatalytic mechanism

**Figure 14:**
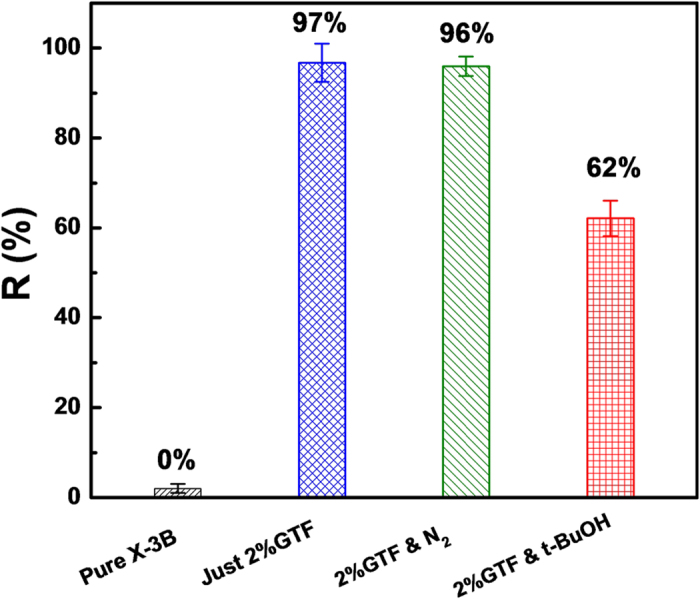
Effect of free radical scavengers on the degradation of X-3B under visible light irradiation.

During the photocatalysis, targeted organic pollutant could mainly be degraded by h^+^, hydroxyl radical (·OH) and superoxide radical (·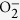) derived from TiO_2_ under irradiation^59^,^60^. The main active species produced in the photocatalysis of 2%GTF and their roles in photocatalytic reaction process were explored by adding different radical scavengers into the system, displayed in Fig. 14.

As shown in Fig. 14, the control sample of pure X-3B solution, in the absence of photocatalysts, did not show any degradation under the light irradiation, excluding the possibility of thermal effects on X-3B degradation. In the presence of 2%GTF, the reactor with X-3B solution was sealed and purged with nitrogen in advance to remove dissolved oxygen in the solution. Thus, the generation of ·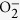 would be suppressed under the irradiation. There was only a slightly decrease, about 1%, in removal rate of X-3B without the presence of ·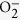, indicating that the contribution of ·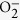 during the photodegradation of X-3B was very small. In addition, as a scavenger for ·OH, 1 mmol/L of tertiary butanol (t-BuOH) was added into the X-3B solution to ensure the non-availability of ·OH for photodegradation. After the same time irradiation, the degradation ratio of X-3B was reduced from 97% to 62%, demonstrating that ·OH contributed more than ·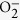 to the photocatalytic reaction. According to the above results, it was reasonable to deduce that about 2/3 of the X-3B in the solution was degraded through the oxidation by h^+^ generated from TiO_2_ under irradiation.

In a word, the photodegradation of X-3B over 2%GTF was driven mainly by the participation of h^+^, with ·OH playing a secondary role and ·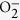 contributing to a much lesser extent.

**Figure 15:**
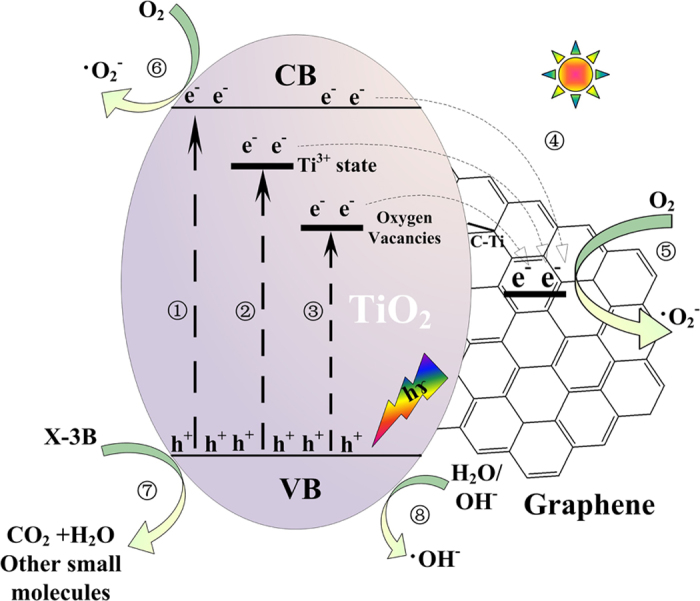
Schematic mechanism illustrations of photocatalysis for 2%GTF composite under visible light irradiation.

The schematic mechanism illustration of the photodegradation process of X-3B over 2%GTF is summarized in Fig. 15.

Under light irradiation, electrons (e^−^) were excited from the valence band (VB) to the conduction band (CB) of TiO_2_, leaving holes (h+) with positive charge in the VB. In the absence of acceptor, the recombination of e^−^ and h^+^ would take place immediately. The oxygen vacancy and Ti^3+^ existed in the bandgap of TiO_2_ in the forms of O_V_^+^, [O_V_·Ti^3+^]^+^ and [O_V_: 2Ti^3+^]^0 ^61. It was reported by Komaguchi *et al*.^62^ that the neutral [O_V_: 2Ti^3+^]^0^ tended to transform to [O_V_·Ti^3+^]^+^ in the presence of O_2_ and light irradiation. The oxygen vacancy color center could be moved up by this transformation, decreasing the visible-light activities. In addition, the formation of C-Ti bond could not only further reduce the bandgap of TiO_2_, realizing the photocatalytic response of 2%GTF in the visible light region, but also improve the transfer efficiency of e^−^ from TiO_2_ to graphene, inhibiting the recombination and prolonging the life-times of separated carries. The electron transfer between TiO_2_ and graphene is presented by


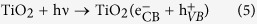



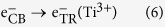



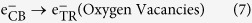



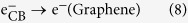


The electrons in the CB of TiO_2_ and graphene would react with oxygen to produce ·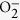 to degrade the pollutants. The process of ·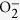 production can be expressed by the following reactions, which contributed a little to the degradation of X-3B, judged from the above detection of active species.


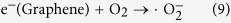



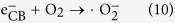



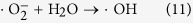


According to the detection of active species produced in the photocatalysis, h^+^ and ·OH played the primary and secondary roles in the photodegradation of X-3B, respectively. The transfer of e^−^ elongates the lifetime of holes in the VB of TiO_2_ and enables their reaction with H_2_O molecules to generate ·OH that can oxidize the X-3B directly. The process can be illustrated by the following equations:


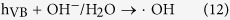



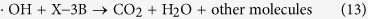



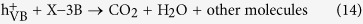


## Conclusions

In conclusion, a brand new approach, force-spinning combined with water vapor annealing method has been developed to fabricate GTF with different graphene contents. According to the characterization results and the analysis of heat treatment process, the as-prepared photocatalysts were subtly designed in such a way that the reduction of GO, the crystallization of TiO_2_, the formation of C-Ti bond between graphene and TiO_2_, and the self-doping of Ti^3+^ and oxygen vacancies could all be achieved in one step. The synergistic effects among great adsorptivity, broadband spectral response, and high charge separation and transportation rates contributed to the efficient photocatalysis in the degradation of X-3B over 2%GTF, significantly superior to PTF and the benchmark photocatalyst of P25 under both UV and visible light irradiation. Based on the detection of active species produced in photocatalysis, the involved mechanisms were also discussed in detail. This work represents a promising way in the preparation of highly efficient graphene/TiO_2_ composite photocatalysts and promote their practical application in environmental remediation and energy storage.

## Methods

### Chemicals and materials

Titanium tetrabutoxide (TBOT), ethyl acetoacetate (EAcAc), tetrahydrofuran (THF), concentrated sulfuric acid (H_2_SO_4_), Potassium Permanganate (KMnO_4_) and hydrogen peroxide (H_2_O_2_) solution were purchased from Sinopharm International Company, Ltd. (Shanghai, China). Graphite powder was obtained from Aladdin Industrial Corporation. (Shanghai, China). Reactive Brilliant Red X-3B was purchased from Shanghai Dyestuff Chemical Plant. All the chemical reagents were of analytical grade and used without further purification. All aqueous solutions were prepared using deionized (DI) water.

### Preparation of graphene oxide

Graphene oxide (GO) nanosheets were synthesized by exfoliating natural graphite powder via using a new method modified from the Hummers and Offeman method^63^. The reduced GO was denoted as RGO. Firstly, 0.5 g of graphite powder and 0.5 g of NaNO_3_ were added to the 23 mL concentrated H_2_SO_4_ cooled (0 °C) with ice bath under vigorous mechanical stirring. Secondly, 3 g of KMnO_4_ was slowly added and the temperature of the above mixture was cooled down to 35 °C and kept this temperature for 1 h. Then 40 mL of DI water was slowly added into the mixture and the temperature was increased to 90 °C and kept constant for 30 min. Finally, 100 mL of DI water was poured quickly into the system so as to terminate the reaction and 5 mL of 10% hydrogen peroxide was added to reduce the residual permanganate and manganese dioxide. The reaction products were separated and washed repeatedly by centrifugation with DI water to adjust the pH of the supernatant to neutral. Then the products were re-dispersed in water by ultrasonic treatment for 30 min to achieve a light-brown solution, namely graphene oxide dispersion. To avoid the agglomeration of GO, the aqueous dispersion was dried by a freeze dryer (UNICRYO MC2, UNIEQUIP, Germany).

### Preparation graphene/TiO_2_ fibers photocatalysts

**Figure 16:**
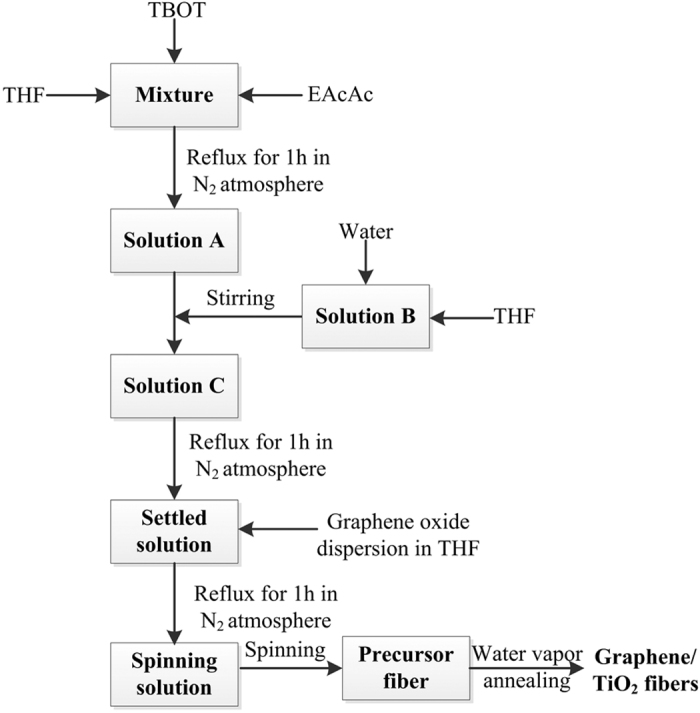
Schematic Diagram of fabricating graphene/TiO_2_ Fiber.

The procedure of preparing graphene/TiO_2_ continuous fibers is summarized in Fig. 16. The precursor fibers were prepared by a sol-gel method combined with centrifugal spinning. TBOT and EAcAc served as the source of Ti and chelating agent, respectively. A mixed solution of 1.11 mL EAcAc/19.8 mL TBOT/26.2 mL THF (1/17.8/23.6 by volume) was refluxed under N_2_ atmosphere for 1 h, to eliminate the interference of other gases in the air, to form solution A. Solution B which was composed of 1.59 mL water (0.79% by volume) dissolved in 200 mL THF, was dropwise added into solution A under stirring to prepare solution C, followed by refluxing for 1 h. Then GO dispersion in THF with different amounts of GO was added into aforementioned settled solution. The obtained sol was then rotary evaporated to prepare the spinning solution with the viscosity of 5 Pa·s. The spinning solution was finally spinned by using a lab-made centrifugal spinning apparatus into long precursor fibers. The as-prepared precursors were annealed in a tube furnace under steam ambient at 500 °C by temperature programmed process. The properties of above composite fibers were compared with those of P25 and the pure TiO_2_ fibers, synthesized by the same method as xGTF, while in the absence of GO and denote and denoted as PTF. The precursor of PTF was referred as PPTF.

### Characterization of prepared photocatalysts

The field-emission scanning electron microscopy (FESEM, JSM-6700F, JEOL) and high-resolution analytical transmission electron microscopy (HRTEM, JEM-2100F, JEOL, at an accelerating voltage of 200 kV) were employed to observe the morphology and structure of as-prepared fibers. The X-ray diffraction (XRD) patterns were obtained on a D/max-γA X-ray diffractometer (Rigaku, Japan), operated at 40 kV and 70 mA, using Cu K_α_ radiation (λ = 0.154178 nm) and at a scanning ratio of 10° min in 2θ ranging from 3° to 70°. The crystallite size of PTF and xGTF was calculated from the anatase (101) diffraction peaks by the Scherrer’s formula: D = 0.9λ/cosθ, where 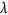 and θ were the average wavelength of the X-ray radiation and Bragg angle, respectively. B was the corrected full peak width at half maximum given by: B^2^ = B_m_^2^ − B_s_^2^, where B_m_ and B_s_ were the measured half-width and instrumental broadening measured from a standard sample of α-silicon (99.9999%)^64^, respectively, and B_s_ was found to be about 0.03 herein. The Fourier transform infrared spectroscopy (FT-IR) spectra were recorded using an Avatar 370 spectrometer (Thermo Nicolet, U.S.). X-ray photoelectron spectroscopy (XPS, ESCALAB 250, Thermo Electron Corp., UK) with an Al K_*α*_ source and a charge neutralizer, was used to detect the changes of the O1s, C1s, and Ti2p binding energies in the samples. All the XPS spectra were calibrated to the C 1 s peak at 284.6 eV. Raman measurement was carried out with a Raman Spectrometer (Renishaw Invia Reflex, Britain) with a 514 nm Ar-ion laser. A liquid nitrogen-cooled Charge Couple Device was employed for Raman spectral detection. The nitrogen adsorption in a Quadrasorb SI-MP system (Quantachrome, U.S.) was used to analyze the BrunauerEmmet-Teller (BET) specific surface area of the fiber photocatalysts. All samples were degassed at 393 K for 8 h in a vacuum prior to BET measurements. Using a multipoint BET method based on the adsorption data in the relative pressure (p/p_0_) range of 0.05–0.3, the BET specific surface area of the fiber was determined. The N_2_ isotherm at 77 K was used to calculate the pore size distribution by using DFT (Density Functional Theory) method^65^. The software QuadraWin supplied by Quantachrome Instruments was used during the measurement. DFT which is based on a molecular-statistical approach, was not applied over a confined range of relative pressure or pore sizes, but over the whole range of the isotherm. The pore size distribution was calculated by fitting the theoretical set of adsorption isotherms which were evaluated for different pore sizes, to the experimental results. The N_2_ adsorption volume at the relative pressure (p/p_0_) of 0.991 was used to determine the pore volume and the average pore size. Solid-state UV-vis diffuse reflectance spectra (DRS) were obtained at room temperature and in air by means of a UV-vis spectrophotometer (UV-3100, Shimadzu, Japan) equipped with an integrating sphere using BaSO_4_ (Shimadzu, Japan) as background. The samples were analyzed using an ESCALAB 250 X-ray photoelectron spectrometer (Thermo Electron Corp., UK) with an Al K_α_ source and a charge neutralizer, and all the spectra were calibrated to the C 1s peak at 284.6 eV. The photoluminescence (PL) spectra were measured by using a F4600 fluorescence spectrophotometer (Hitachi, Japan) with scan range from 220 to 900 nm and excitation wavelength at 375 nm.

### Photocatalytic activity studies

All the photocatalysis experiments were performed in a quartz chamber cooled with circulating water, fitted with a 250W high-pressure mercury lamp (λ_max_ = 365 nm), and a 1000 W Xe lamp with a filter to cut off the short wavelength components (λ < 420 nm) as the UV and visible light source, respectively. The irradiation intensity of the UV lamp (15 W m^−2^) at the surface of dye solution was measured with digital illuminometer (TN-2340, Taiwan). Before irradiation, 0.4 g of the prepared photocatalysts were placed into the chamber containing 100 mL of 30 mg L^−1^ X-3B solution was magnetically stirred for 30 min in the dark to establish the adsorption/desorption equilibrium between X-3B and the photocatalyst surface. Next, the reaction mixture was illuminated under UV or visible light for the entire time span of experiment. At regular intervals, samples of about 5 mL in volume were taken and filtrated through a 0.45 μm syringe filter. The extent of X-3B removal was determined by measuring the absorbance on a UV-vis 1601 spectrophotometer (Shimadzu, Japan) at 540 nm. The removal rate of the dyes was calculated by


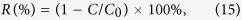


where C_0_ and C were the concentration of dyes at reaction times 0 and t, respectively.

## Additional Information

**How to cite this article**: Zhang, Q. *et al*. Advanced Fabrication of Chemically Bonded Graphene/TiO_2_ Continuous Fibers with Enhanced Broadband Photocatalytic Properties and Involved Mechanisms Exploration. *Sci. Rep.* **6**, 38066; doi: 10.1038/srep38066 (2016).

**Publisher's note:** Springer Nature remains neutral with regard to jurisdictional claims in published maps and institutional affiliations.

## Figures and Tables

**Table 1:** Grain size and FWHM of PTF and xGTF (x = 0.5%, 1% and 2%).

Sample	FWHM	Grain Size/nm
PTF	0.370	22.9
0.5%GTF	0.383	22.1
1%GTF	0.606	13.6
2%GTF	0.708	12.6

**Table 2:** Textural parameters of PTF and xGTF (x = 0.5%, 1% and 2%).

Samples	BET Surface Area (m^2^/g)	Pore Volume (cm^3^/g)	Pore Diameter (nm)
0.5%GTF	45.648	0.185	5.300
1%GTF	72.370	0.188	9.416
2%GTF	86.772	0.204	11.680
PTF	44.834	0.177	3.627
